# Supplementary material for: Toxicity of TiO2 Nanoparticles: Validation of Alternative Models
Source: Int J Mol Sci. 2020 Jul 9;21(14):4855. doi: 10.3390/ijms21144855 (PMC7402355; doi:10.3390/ijms21144855)
Supplement: Supplementary file 1 [file ijms-21-04855-s001.zip › Supp Data 2.pdf]

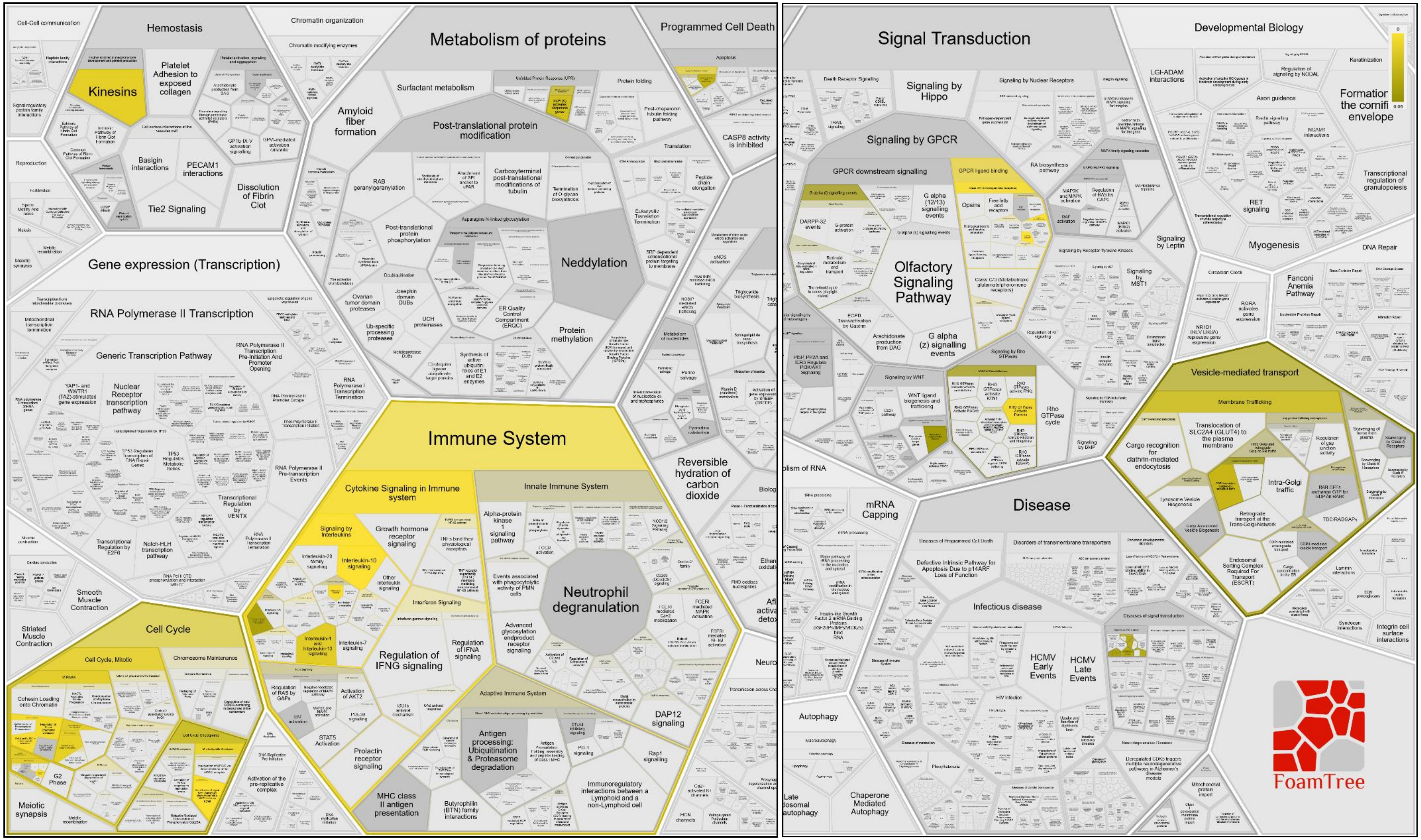

Figure S2: Reactome analysis of the 18 common genes (by Reactome Database, from the gene list, projected to human, pValue < 0,05)

Table SII. Dysregulated pathways (18 dysregulated genes analyzed by Reactome Database, projected to human)

Pathway name	Curated found	Curated total	Entities found	Entities total	Entities ratio	Entities pValue	Reactions found	Reactions total	Reactions ratio
**Chemokine receptors bind chemokines**	4	57	4	103	0.005	3.64E-5	*1*	*19*	*0.002*
**Interleukin-10 signaling**	4	86	4	171	0.008	2.53E-4	*1*	*15*	*0.001*
Peptide ligand-binding receptors	5	211	5	413	0.02	8.32E-4	*2*	*76*	*0.006*
**Mitotic Prometaphase**	4	211	4	276	0.013	1.5E-3	*8*	*20*	*0.002*
**Class A/1 (Rhodopsin-like receptors)**	6	462	6	814	0.04	3.1E-3	*4*	*152*	*0.012*
**Amplification of signal from unattached kinetochores via a MAD2 inhibitory signal**	3	94	3	166	0.008	3.33E-3	*4*	*4*	*0*
**Amplification of signal from the kinetochores**	3	94	3	166	0.008	3.33E-3	*4*	*4*	*0*
**EML4 and NUDC in mitotic spindle formation**	3	121	3	184	0.009	4.43E-3	*1*	*5*	*0*
**Resolution of Sister Chromatid Cohesion**	3	134	3	188	0.009	4.7E-3	*4*	*8*	*0.001*
**RHO GTPases Activate Formins**	3	149	3	191	0.009	4.91E-3	*3*	*27*	*0.002*
**Mitotic Spindle Checkpoint**	3	110	3	200	0.01	5.58E-3	*4*	*7*	*0.001*
**Separation of Sister Chromatids**	3	194	3	208	0.01	6.22E-3	*2*	*8*	*0.001*
**Mitotic Anaphase**	3	208	3	223	0.011	7.52E-3	*2*	*11*	*0.001*
**GPCR ligand binding**	6	652	6	1,021	0.05	9.22E-3	*4*	*179*	*0.014*
**Mitotic Metaphase and Anaphase**	3	211	3	280	0.014	1.39E-2	*2*	*12*	*0.001*
**M Phase**	4	391	4	563	0.027	1.81E-2	*10*	*70*	*0.006*
**Interleukin-4 and Interleukin-13 signaling**	3	211	3	339	0.016	2.29E-2	*2*	*46*	*0.004*
