# Supplementary material for: Toxicity of TiO2 Nanoparticles: Validation of Alternative Models
Source: Int J Mol Sci. 2020 Jul 9;21(14):4855. doi: 10.3390/ijms21144855 (PMC7402355; doi:10.3390/ijms21144855)
Supplement: Supplementary file 1 [file ijms-21-04855-s001.zip › Supp Data 1.pdf]

Table S1. 40 most deregulated genes in each exposition methods

In-vivo lung			*In-vitro* ALI			*In-vitro* submerged		
Gene Name	FC	FDR p-value	Gene Name	FC	FDR p-value	Gene Name	FC	FDR p-value
**Up-regulated genes**								
Retnla	11,57	6,62E-06	Ddit4	2,31	2,17E-02	Cxcl2	37,2	1,99E-07
Prss30	11,33	3,66E-07	Ceacam19	2,30	3,91E-03	Tac4	27,8	8,71E-08
Orm1	10,20	4,3E-06	Chac1	2,20	2,28E-03	Slpi	15,6	1,08E-05
Lcn2	9,28	2,66E-06	**Ccl4**	2,15	3,28E-03	Tbkbp1	14,9	1,69E-06
Slc26a4	8,57	2,36E-06	Zfp668	1,95	2,28E-03	Slpil3	14,7	3,01E-06
Ccl1	8,15	6,62E-06	Nab2	1,92	2,98E-03	Slpi	13,3	1,59E-05
Cxcl5	6,21	2,18E-05	Fam129b	1,90	3,97E-03	Phf19	13,2	1,99E-07
Bpifb1	6,07	1,12E-05	Pcdh15	1,88	2,87E-02	Gdf15	13,2	4,50E-07
Ccl2	5,77	2,82E-05	Ganc	1,84	2,32E-02	Socs3	11,7	3,45E-07
**Ccl7**	5,61	6,8E-05	Slc6a6	1,79	4,22E-02	Timm8a2	11,6	6,32E-07
Ccl12	5,54	5,58 E-04	Csrnp1	1,78	1,00E-02	Cox6a2	11,0	4,20E-05
Cd177	5,32	8,53E-05	Nr3c2	1,76	2,18E-02	Syt8	10,8	3,08E-07
Lpo	5,32	1,85 E-04	Abcf3	1,74	1,67E-02	C3	10,6	2,18E-06
Spp1	5,13	4,36 E-04	Wisp1	1,74	7,88E-03	Jak3	10,5	1,72E-06
Rhbdl2	4,96	1,08E-05	Wdr81	1,74	1,88E-02	Cox6a2	10,2	9,39E-05
Noxo1	4,91	1,28 E-04	**Osgin1**	1,74	1,87E-02	Csf3	10,0	2,90E-07
Defb5	4,82	1,08E-05	Aldoa	1,73	3,10E-02	Egr2	9,6	1,14E-06
Itih1	4,65	2,66E-06	Per1	1,73	7,88E-03	Maff	9,6	2,38E-07
Mab21l3	4,44	1,26E-03	BU760410	1,72	4,52E-02	Gtsf1	9,0	1,30E-05
CB548020	4,43	4,63E-06	Cass4	1,71	8,33E-03	Sohlh2	9,0	3,54E-07
**Down-regulated genes**								
Nr1d1	8,74	9,25E-06	Myc	3,04	5,12E-04	Calcr	43,09	3,45E-07
Hspa1b	6,87	6,72E-04	Ccng2	2,31	5,12E-04	Dynlt3	41,71	8,71E-08
Dbp	4,09	3,32E-05	Id1	2,29	5,12E-04	DQ813342	33,78	8,71E-08
Ear11	2,86	4,61E-05	Dusp6	2,27	6,91E-03	Cyp4a8	30,39	1,42E-06
Cyp2a3	2,85	2,14E-03	Txnip	2,19	1,15E-03	Tp53inp1	23,98	2,04E-06
Gnat1	2,75	1,15E-04	Cdc42ep3	2,17	6,76E-04	Pla2g2d	22,44	2,09E-06
Ccdc116	2,75	1,59E-03	Arrdc3	2,10	1,07E-02	March1	20,73	3,47E-07
Dlk1	2,66	1,80E-02	BG664185	2,01	6,00E-03	Ar	20,45	6,85E-06
Tac1	2,57	3,96E-04	Klf10	1,89	8,46E-03	Mki67	18,57	5,06E-07
Nr1d2	2,52	3,87E-05	FQ233077	1,87	1,78E-02	Igf1	17,25	2,75E-07
RGD1566226	2,50	7,52E-03	Hist1h4b	1,84	1,16E-02	Agbl4	17,12	1,91E-07
Nr4a1	2,41	4,02E-02	Zmat1	1,82	5,62E-03	Mid1ip1	16,60	3,76E-07
Amigo2	2,41	1,95E-04	RGD1562660	1,82	2,04E-02	Bclaf1	16,58	4,52E-07
Dll1	2,37	1,35E-03	Tp53inp1	1,81	1,23E-02	Olr1	16,44	8,51E-07
Npr3	2,35	3,64E-04	Ucp3	1,79	1,16E-02	Bche	16,29	4,93E-07
Gpihbp1	2,28	5,67E-04	Cytip	1,79	8,33E-03	Dmd	16,20	8,71E-07
Rnase17	2,27	1,23E-03	Rnf149	1,79	2,04E-02	Mitf	15,78	1,63E-05
Nrarp	2,23	2,86E-04	Ifit2	1,78	9,43E-03	Dmxl2	15,54	5,96E-06
Alox15	2,20	1,10E-03	Ns5atp9	1,75	3,10E-02	Tspan7	14,70	2,06E-07
Kcna6	2,19	7,23E-03	Ccdc132	1,73	9,74E-03	FQ234973	14,62	1,78E-07

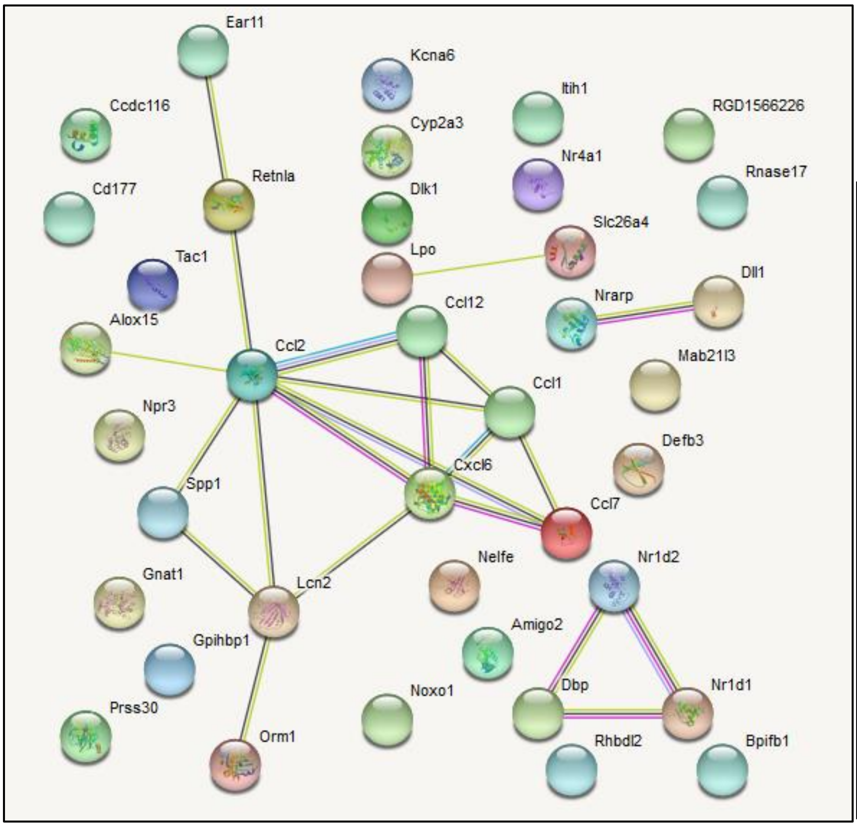

Network Stats

Number of nodes: 39

Number of edges: 22

Average node degree: 1.13

Avg. local clustering coefficient: 0.385

Expected number of edges: 3

PPI enrichment p-value: 8.73e-14

Rq: Your network has significantly more interactions than expected. Such an enrichment indicates that the proteins are at least partially biologically connected, as a group.

KEGG Pathways	Pathway description	count in gene set	false discovery rate
rno04657	IL-17 signaling pathway	5 of 90	2.27e-05
rno04062	Chemokine signaling pathway	5 of 168	0.00022
rno05323	Rheumatoid arthritis	3 of 82	0.0054
rno04060	Cytokine-cytokine receptor interaction	4 of 217	0.0054
rno05144	Malaria	2 of 51	0.0290

GO-term	description	count in gene set	false discovery rate
GO:0071396	cellular response to lipid	8 of 359	0.00014
GO:0006952	defense response	9 of 487	0.00014
GO:0071310	cellular response to organic substance	11 of 1129	0.00081
GO:0071383	cellular response to steroid hormone stimulus	5 of 148	0.0012
GO:0033993	response to lipid	9 of 772	0.0012
GO:0006955	immune response	7 of 386	0.0012
GO:0006954	inflammatory response	6 of 250	0.0012
GO:0032101	regulation of response to external stimulus	6 of 297	0.0017
GO:0070098	chemokine-mediated signaling pathway	3 of 30	0.0026
GO:0009617	response to bacterium	6 of 335	0.0026
GO:0065007	biological regulation	20 of 4801	0.0029
GO:0048545	response to steroid hormone	6 of 353	0.0029
GO:2000425	regulation of apoptotic cell clearance	2 of 4	0.0030
GO:0071407	cellular response to organic cyclic compound	6 of 385	0.0030
GO:0050789	regulation of biological process	19 of 4461	0.0030
GO:0042592	homeostatic process	8 of 790	0.0030
GO:0010332	response to gamma radiation	3 of 37	0.0030
GO:0002690	positive regulation of leukocyte chemotaxis	3 of 37	0.0030
GO:0048583	regulation of response to stimulus	11 of 1609	0.0031
GO:0032879	regulation of localization	10 of 1378	0.0033

Figure S1.A. **Interactions, KEGG pathways and GO biological process** (20 most deregulated GO-term) analysis of the 20 more up-regulated and 20 down-regulated DEG **in NR8383 *in-vitro*** after TiO2 NP exposure (analyzed by String 11.1 Database).

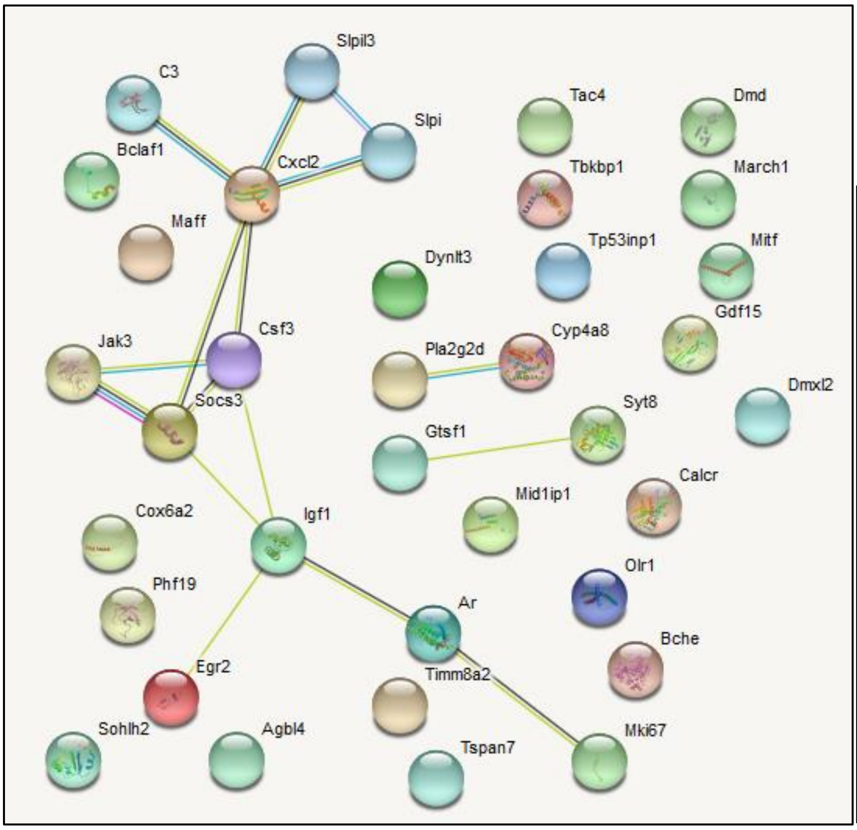

Network Stats

Number of nodes: 36

Number of edges: 16

Average node degree: 0.889

Avg. local clustering coefficient: 0.316

Expected number of edges: 5

PPI enrichment p-value: 3.39e-05

Rq: Your network has significantly more interactions than expected. Such an enrichment indicates that the proteins are at least partially biologically connected, as a group.

**GO-term**	**description**	**count in gene set**	**false discovery rate**
GO:2000112	regulation of cellular macromolecule biosynthetic process	9 of 1309	0.0104
GO:1901700	response to oxygen-containing compound	9 of 1217	0.0104
GO:1901654	response to ketone	5 of 249	0.0104
GO:0080090	regulation of primary metabolic process	12 of 2165	0.0104
GO:0065009	regulation of molecular function	9 of 1462	0.0104
GO:0065007	biological regulation	18 of 4801	0.0104
GO:0060255	regulation of macromolecule metabolic process	12 of 2174	0.0104
GO:0051384	response to glucocorticoid	4 of 227	0.0104
GO:0051173	positive regulation of nitrogen compound metabolic process	8 of 1184	0.0104
GO:0051172	negative regulation of nitrogen compound metabolic process	7 of 854	0.0104
GO:0051171	regulation of nitrogen compound metabolic process	11 of 2091	0.0104
GO:0050896	response to stimulus	16 of 3604	0.0104
GO:0050794	regulation of cellular process	16 of 4191	0.0104
GO:0050789	regulation of biological process	17 of 4461	0.0104
GO:0048585	negative regulation of response to stimulus	6 of 596	0.0104
GO:0048545	response to steroid hormone	5 of 353	0.0104
GO:0048522	positive regulation of cellular process	12 of 2201	0.0104
GO:0048519	negative regulation of biological process	11 of 2096	0.0104
GO:0048518	positive regulation of biological process	13 of 2425	0.0104
GO:0048513	animal organ development	9 of 1420	0.0104

Figure S1.B. **Interactions and GO biological process** (20 most deregulated GO-term) of the 20 more up-regulated and 20 down-regulated DEG **in lung rat exposed *in-vivo*** to TiO2 NP (analyzed by String 11.1 Database).

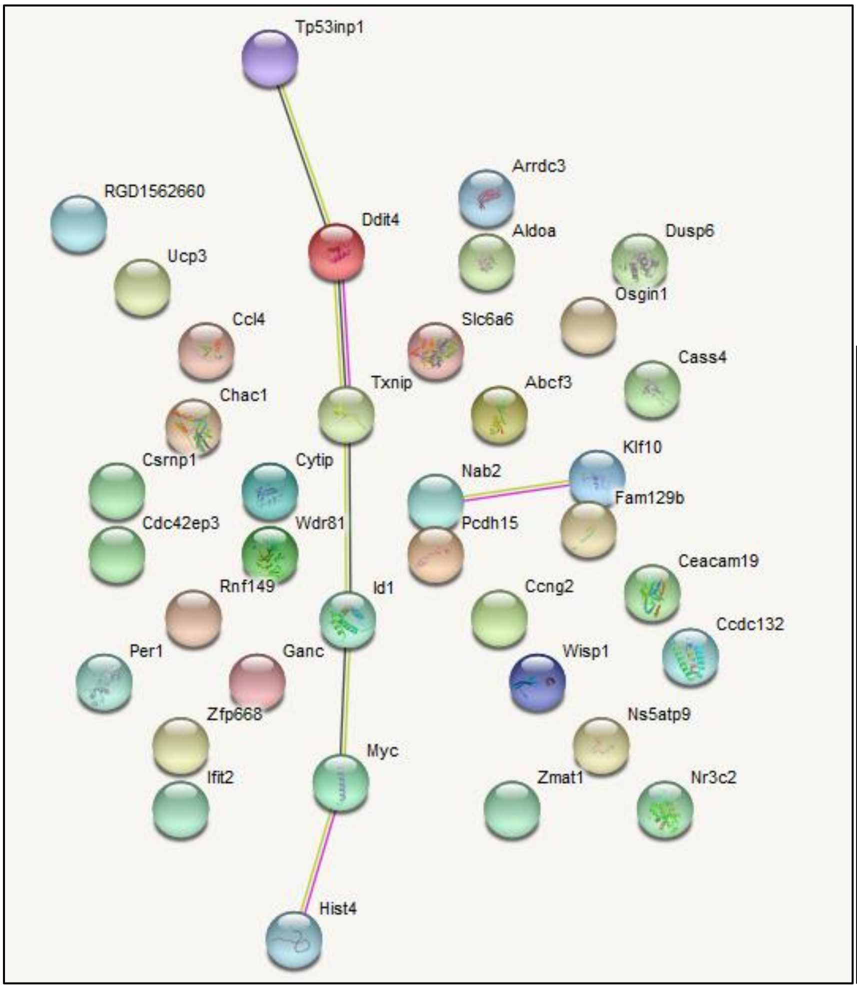

Network Stats

Number of nodes: 37

Number of edges: 6

Average node degree: 0.324

Avg. local clustering coefficient: 0.108

Expected number of edges: 2

PPI enrichment p-value: 0.0412

Rq: Your network has significantly more interactions than expected. Such an enrichment indicates that the proteins are at least partially biologically connected, as a group.

**GO-term**	**description**	**count in gene set**	**false discovery rate**
GO:0080090	regulation of primary metabolic process	14 of 2165	0.0018
GO:0060255	regulation of macromolecule metabolic process	13 of 2174	0.0018
GO:0051172	negative regulation of nitrogen compound metabolic process	9 of 854	0.0018
GO:0051171	regulation of nitrogen compound metabolic process	13 of 2091	0.0018
GO:0048523	negative regulation of cellular process	13 of 1880	0.0018
GO:0045892	negative regulation of transcription, DNA-templated	6 of 364	0.0018
GO:0034654	nucleobase-containing compound biosynthetic process	10 of 943	0.0018
GO:0031324	negative regulation of cellular metabolic process	10 of 921	0.0018
GO:0031323	regulation of cellular metabolic process	14 of 2217	0.0018
GO:0010629	negative regulation of gene expression	7 of 531	0.0018
GO:0010605	negative regulation of macromolecule metabolic process	9 of 914	0.0018
GO:0006950	response to stress	11 of 1597	0.0018
GO:0006351	transcription, DNA-templated	8 of 733	0.0018
GO:0051253	negative regulation of RNA metabolic process	6 of 413	0.0024
GO:0051101	regulation of DNA binding	3 of 43	0.0025
GO:0034645	cellular macromolecule biosynthetic process	9 of 1128	0.0027
GO:0050896	response to stimulus	16 of 3604	0.0028
GO:0010033	response to organic substance	11 of 1770	0.0030
GO:0090304	nucleic acid metabolic process	9 of 1193	0.0033
GO:0051782	negative regulation of cell division	2 of 7	0.0033

Figure S1.C. Interactions and GO biological process (20 most deregulated GO-term) of the 20 more up-regulated and 20 down-regulated DEG **in NR8383 after TiO2 NP ALI Vitrocell Cloud ® exposure**, (analyzed by String 11.1 Database).
